# Vv-AMP1, a ripening induced peptide from *Vitis vinifera *shows strong antifungal activity

**DOI:** 10.1186/1471-2229-8-75

**Published:** 2008-07-08

**Authors:** Abré de Beer, Melané A Vivier

**Affiliations:** 1 Institute for Wine Biotechnology, Department of Viticulture and Oenology, Faculty of AgriSciences, Stellenbosch University, Stellenbosch 7600, South Africa

## Abstract

**Background:**

Latest research shows that small antimicrobial peptides play a role in the innate defense system of plants. These peptides typically contribute to preformed defense by developing protective barriers around germinating seeds or between different tissue layers within plant organs. The encoding genes could also be upregulated by abiotic and biotic stimuli during active defense processes. The peptides display a broad spectrum of antimicrobial activities. Their potent anti-pathogenic characteristics have ensured that they are promising targets in the medical and agricultural biotechnology sectors.

**Results:**

A berry specific cDNA sequence designated *Vv-AMP1*, *Vitis vinifera *antimicrobial peptide 1, was isolated from *Vitis vinifera*. *Vv-AMP1 *encodes for a 77 amino acid peptide that shows sequence homology to the family of plant defensins. *Vv-AMP1 *is expressed in a tissue specific, developmentally regulated manner, being only expressed in berry tissue at the onset of berry ripening and onwards. Treatment of leaf and berry tissue with biotic or abiotic factors did not lead to increased expression of *Vv-AMP1 *under the conditions tested. The predicted signal peptide of Vv-AMP1, fused to the green fluorescent protein (GFP), showed that the signal peptide allowed accumulation of its product in the apoplast. Vv-AMP1 peptide, produced in *Escherichia coli*, had a molecular mass of 5.495 kDa as determined by mass spectrometry. Recombinant Vv-AMP1 was extremely heat-stable and showed strong antifungal activity against a broad spectrum of plant pathogenic fungi, with very high levels of activity against the wilting disease causing pathogens *Fusarium oxysporum *and *Verticillium dahliae*. The Vv-AMP1 peptide did not induce morphological changes on the treated fungal hyphae, but instead strongly inhibited hyphal elongation. A propidium iodide uptake assay suggested that the inhibitory activity of Vv-AMP1 might be associated with altering the membrane permeability of the fungal membranes.

**Conclusion:**

A berry specific cDNA clone, *Vv-AMP1*, was isolated and characterized and shown to encode a plant defensin. Recombinant Vv-AMP1 displayed non-morphogenic antifungal activity against a broad spectrum of fungi, probably altering the membrane permeability of the fungal pathogens. The expression of this peptide is highly regulated in *Vitis vinifera*, hinting at an important defense role during berry-ripening.

## Background

Plants are constantly subjected to microbial attack, especially phytopathogenic fungi and use various defense strategies to protect themselves against disease. These defenses include the strengthening of the physical cell wall barriers [1] and the production of chemical and proteinaceous antimicrobial compounds [2-6]. Over the last 15 years it has become evident that small, basic, cysteine-rich peptides also form part of the overall defense of plants against phytopathogens, contributing significantly to the innate immunity of plants [7-10]. It has been suggested that all plants possess such a peptide defense system [8]. The peptides range from 2–9 kDa in size and the best characterized examples are the thionins and defensins [7,8,10-15]. When first isolated, defensins were classified as γ-thionins, but were later renamed to plant defensins due to their structural and functional similarities to insect and human defensins [16-18].

Plant defensins are a family of basic, cysteine-rich peptides of between 45–54 amino acids in size. Structurally they consist of one α-helix and one β-sheet, comprising three antiparallel β-strands, and stabilized by the formation of disulfide bridges between the cysteine residues [19-22]. Although plant defensins are structurally conserved, their overall homology at the amino acid level is low. However, most plant defensins contain eight cysteine residues linked by four disulfide bridges, an aromatic residue at position 11, two glycines at positions 13 and 34 and a glutamate at position 29 (numbering according to Rs-AFP1 [23]).

Most plant defensins exhibit some antimicrobial activity, inhibiting the growth of fungi, oomycetes and gram positive bacteria *in vitro*. The exact mechanisms underlying the antifungal activity exerted by plant defensins are not known, but there is evidence that plant defensins bind to a specific receptor in the fungal membrane, being sphingolipids, rather than random binding and integration into the phospholipid bilayer of the fungal membranes [24-31]. Other biological activities such as proteinase and α-amylase inhibition [22,32,33], metal tolerance [34], as well as the inhibition of protein translation and HIV proliferation have also been reported for some of the isolated plant defensins [35-37].

The majority of defensins have been isolated from plant seeds [35-43], but defensins have also been isolated from leaves [23,44], flowers [45-49], tubers [50], seedpods [38], as well as from fruits [51-53]. Although plant defensins play an important role in the preformed defense, some members of the defensin family are also upregulated upon pathogen attack or by environmental stimuli, while the expression of others are strictly developmentally regulated [34,51,52,54-59].

Here we report the isolation and characterization of the first plant defensin from *Vitis vinifera*. The peptide encoding gene shows a strict tissue-specific and developmentally regulated expression pattern. The peptide is strongly antifungal without inducing morphological changes to the hyphae of the tested fungi, but with an indication of a compromising effect on the fungal membrane. The ripening-specific expression pattern in berries and the strong *in vitro *antifungal characteristics of the isolated peptide draws interest to its possible *in vivo *role in berry defence systems.

## Results

### Isolation and genomic characterization of *Vv-AMP1*

**Figure 1:**
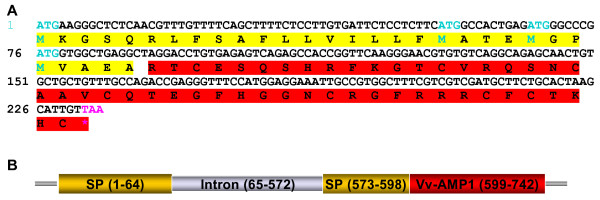
**Gene structure of *Vv-AMP1***. (**A**) The complete coding sequence of *Vv-AMP1 *with its deduced amino acid sequence. The amino acids in yellow represent the signal peptide while red amino acids indicate the mature peptide. (**B**) The genomic structure of the *Vv-AMP1 *gene. Yellow blocks represent the sequence encoding for the signal peptide of Vv-AMP1 and the red block the sequence encoding for the mature Vv-AMP1 peptide. The intron is indicated as a grey block. Numbering inside each block corresponds to the number of base pairs in each section.

Initial screening of the *Vitis vinifera *EST database at The Institute for Genomic Research (TIGR) with the BLAST algorithm yielded only one EST hit, TC69032. Subsequent analyses of the available databases after the completion of the grapevine genome sequence, has yielded other putative defensin sequences (results not shown). Primer design was based on the EST TC69032 and PCR screening of cDNA batches made from grapevine root, leaf and berry material allowed for the isolation of a single complete coding sequence from grape berry cDNA. The sequence was termed *Vitis vinifera *antimicrobial peptide 1 (*Vv-AMP1*), because of its homology to the family of plant defensins. The complete coding sequence of *Vv-AMP1 *is 234 bp in size and encodes for a predicted 77 amino acid peptide, comprising a 30 amino acid signal peptide and a 47 amino acid mature peptide (Figure 1A). The genomic copy isolated for *Vv-AMP1 *is 742 bp in size and comparative analyses with the cDNA sequences revealed that a 508 bp intron interrupts the predicted signal peptide (Figure 1B).

**Figure 2:**
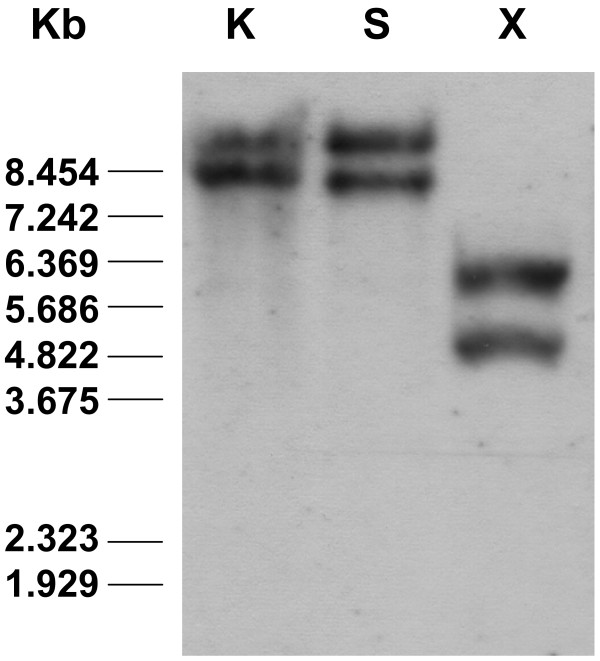
**Southern blot analysis of *Vitis vinifera *genomic DNA to evaluate the genomic organisation of *Vv-AMP1***. *Vitis vinifera *cv. Pinotage genomic DNA was digested with *Kpn*I (K), *Spe*I (S) or *Xba*I (X) and probed with a DIG labeled cDNA copy of *Vv-AMP1*. Lambda DNA digested with *Bst*EII was used as marker (sizes indicated). Each signal indicates a single copy of *Vv-AMP1*.

Southern blot analysis conducted on genomic DNA from cultivar Pinotage revealed the presence of two hybridization signals for *Vv-AMP1 *within the *V. vinifera *genome (Figure 2). BLAST analysis of the grapevine genome sequence at the National Centre for Biotechnological Information (NCBI) identified two possible contig sequences, VV78X055073.5 and VV78X034124.3. Alignment analysis of these two genomic sequences showed that the nucleotide areas upstream and downstream of the *Vv-AMP1 *open reading were similar, suggesting that a single copy of *Vv-AMP1 *is present in the *V. vinifera *genome.

**Figure 3:**
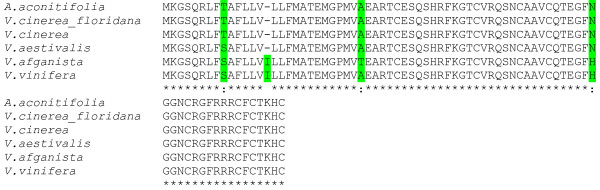
**Alignment analysis of the deduced amino acid sequences of the *Vv-AMP1 *genes isolated from different *Vitis *species**. The major differences are indicated in green, note the additional amino acid at position 15 of the sequences isolated from *V. vinifera and V. afganista *(numbering according to Vv-AMP1 from *V. vinifera*).

Alignment analysis of the genomic sequences of *Vv-AMP1 *isolated from non-vinifera *Vitis *species revealed a high level of homology (93%) at nucleotide level. When the deduced coding sequences for the different *Vv-AMP1 *genes were compared, up to 95% homology was observed, translating into 93% identity at the deduced amino acid level (Figure 3). *Vv-AMP1 *from *V. vinifera *showed the highest homology to the gene amplified from *V. afganista*, sharing 98.7% homology at amino acid level. The deduced amino acid sequences from *V. vinifera *and *V. afganista *differed from the rest by having one additional amino acid in their signal peptide region, with the introduction of isoleucine at position 15 (numbering according to Vv-AMP1 from *V. vinifera*).

### Bioinformatical characterization of the deduced amino acid sequence of Vv-AMP1

**Figure 4:**
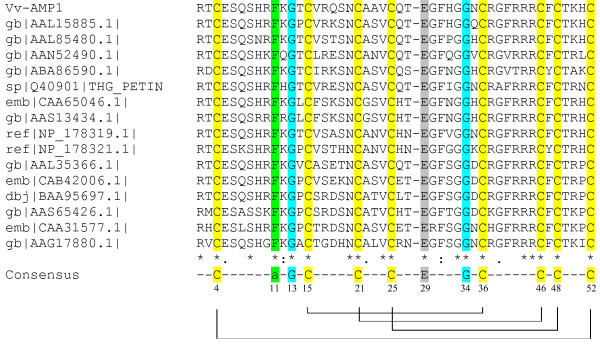
**Amino acid alignment analyses of plant defensins belonging to subfamily B1 and Vv-AMP1 from *Vitis vinifera***. gb|AAL15885.1| putative γ-thionin [*Castanea sativa*]; gb|AAL85480.1| defensin protein 1 [*Prunus persica*]; gb|AAN52490.1| defensin EGAD1 [*Elaeis guineensis*]; gb|ABA86590.1| putative defensin 1 [*Aquilegia olympica*]; sp|Q40901|THG_PETIN γ-thionin homolog PPT precursor [*Petunia inflata*]; emb|CAA65046.1 unnamed protein product [*Capsicum annuum*]; gb|AAS13434.1| defensin [*Nicotiana attenuata*]; ref|NP_178319.1| LCR69/PDF2.2; protease inhibitor [*Arabidopsis thaliana*]; ref|NP_178321.1| LCR68/PDF2.3; protease inhibitor [*Arabidopsis thaliana*]; gb|AAL35366.1| defensin protein precursor [*Capsicum annuum*]; emb|CAB42006.1 γ-thionin [*Lycopersicon esculentum*]; dbj|BAA95697.1| thionin like protein [*Nicotiana tabacum*]; gb|AAS65426.1| Kunitz-type trypsin inhibitor [*Ipomoea batatas*]; emb|CAA31577.1 unnamed protein product [*Solanum tuberosum*]; gb|AAG17880.1| Kunitz trypsin inhibitor protein [*Phaseolus coccineus*]. The consensus sequence common to all defensins is indicated below with numbering according to Rs-AFP1 [23]. The eight cysteines are indicated in yellow and the aromatic residue at position 11 in green. The conserved glycines are indicated in blue and glutamate at position 29 in grey. The gap was introduced to have number agreement with Rs-AFP1. The disulfide bridge organization within the Vv-AMP1 sequence is indicated below the consensus sequence.

BLASTP results and further alignment analysis showed that the deduced amino acid sequence of *Vv-AMP1 *shared high homology to the γ-thionins from *Castanea sativa *and PPT from petunia (Figure 4). Vv-AMP1 also displays the following conserved amino acid residues: an aromatic residue at position 11, two glycine residues at positions 13 and 34 and a glutamate at position 29, as well as the eight cysteine residues at positions 4, 15, 21, 25, 36, 46, 48, 52 present in all plant defensins (numbering according to Rs-AFP1 [23]). Disulfide bridge analysis done with DIpro showed that the eight cysteine residues of Vv-AMP1 are connected by four disulfide bridges (Figure 4).

**Figure 5:**
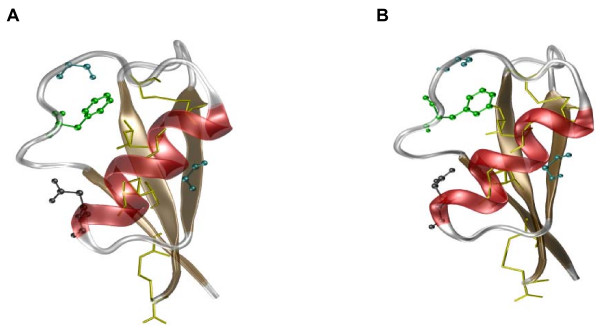
**Comparison of the tertiary structure of Vv-AMP1 from grapevine (A) and hordothionin-γ from barley (B)**. The α-helix and β-sheet structures are represented in red and ochre respectively with the conserved amino acids represented in ball and stick models and colored according to the conserved sequence in Figure 4.

Comparative homology modeling of the deduced amino acid sequence confirmed that the tertiary structure of Vv-AMP1 closely resembled that of hordothionin-γ (1GPT) from barley and had the typical defensin structure consisting of an α-helix and a triple-stranded antiparallel β-sheet, which are organized in a βαββ configuration (Figure 5). The structure is stabilized by intramolecular disulfide linkages between the eight cysteine residues.

### Targeting ability of the putative Vv-AMP1 signal peptide

**Figure 6:**
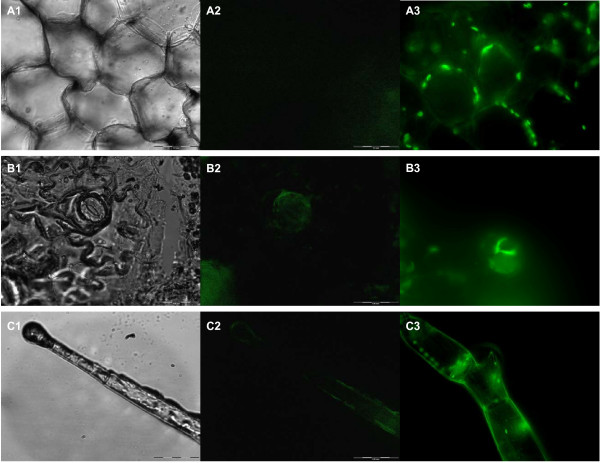
**Localization of GFP in transgenic tobacco as directed by the signal peptide of Vv-AMP1**. Localization of GFP observed in the apoplasts of the following tissues, organs and cells in the leaf petiole: (**A**) cortex; (**B**) the guard cells of the stomata and (**C**) the trichoma. A1, B1 and C1 show light microscopic photos of these various tissues and organs from the untransformed control. A2, B2 and C2 show the auto-fluorescence from these same fields in the untransformed controls, whereas A3, B3 and C3 indicate GFP expression in the apoplastic regions of these structures in the GFP overexpressing lines.

PA-SUB predicted that the signal peptide of Vv-AMP1 directs its product to the apoplastic regions of plant cells. This was confirmed by fusing the Vv-AMP1 signal peptide to GFP under constitutive expression and overexpressing it into tobacco. Inverted fluorescent microscopy conducted on free-hand cross sections of the tobacco leaf petiole revealed that the GFP accumulated in the apoplastic regions (Figure 6).

### Expression profile of *Vv-AMP1 *within *V. vinifera*

**Figure 7:**
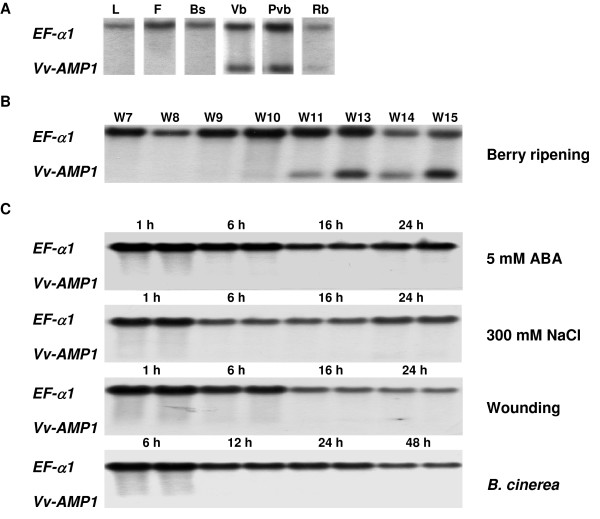
**The expression profile of *Vv-AMP1 *within the grapevine cultivar Pinotage**. (**A**) Northern blot analysis of total RNA isolated from leaf (L) and flower tissue (F) as well as four berry developmental stages: Berry set (Bs), Véraison (Vb), Post-véraison (Pvb), Ripe (Rb). (**B**) Induction of *Vv-AMP1 *by developmental regulation (**C)**. Biotic and abiotic induction studies were conducted on Pinotage leaf tissue. Time points indicate the time of tissue collection after the initiation of each induction experiment. Northern blot signals were detected by probing with a DIG labelled *Vv-AMP1 *cDNA and a *V. vinifera EF-α1 *probe as internal standard. The *Vv-AMP1 *signal hybridized at a molecular weight of 500 bp and the internal standard at 2500 bp.

Our investigation of the expression pattern of *Vv-AMP1 *within grapevine, revealed that this gene is expressed in a tissue-specific manner, being only expressed in berries (Figure 7A). Northern blot analysis on berries in different stages of development and ripening confirmed that the gene is developmentally regulated. *Vv-AMP1 *expression was induced upon berry ripening, starting at véraison, 11 weeks post-flowering (Figure 7B). Expression of *Vv-AMP1 *remained high throughout the rest of the berry ripening stages. Induction studies, conducted on grapevine leaf material, simulating osmotic stress, wounding, pathogen infection with *Botrytis cinerea *as well as treatment with ABA, were unable to induce *Vv-AMP1 *expression (Figure 7C). The experiment was also repeated on pre-véraison berries, but none of the induction stimuli could overcome the developmental regulation (results not shown). On the pre-véraison berries, JA and SA treatments were included without any induction observed (results not shown).

### Recombinant production of Vv-AMP1

**Figure 8:**
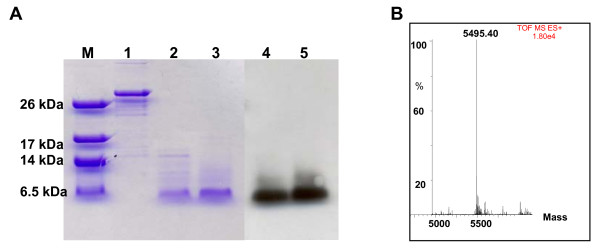
**Size determination and Western blot analysis of the purified recombinant Vv-AMP1 peptide**. (**A**) SDS-PAGE analysis of the GST-fusion protein before and after thrombin treatment; lane M, low molecular weight marker (Sigma, St Louis, USA); lane 1 GST-fusion protein, lane 2 and 3 purified Vv-AMP1 peptide after thrombin digestion and cation exchange chromatography; lanes 4 and 5, Western blot analysis of Vv-AMP1. (**B**) Mass spectrometric analysis of recombinant Vv-AMP1 after separation from the GST-tag using ion exchange chromatography.

Recombinant Vv-AMP1, fused to the GST-tag, was successfully produced in *E. coli *by using the Rosetta gami pLysS expression system. Purification of the recombinant peptide using a glutathione affinity chromatography system (Sigma, St Louis, USA) yielded 5 mg/L purified peptide. The recombinant fusion protein had a size of 31 kDa, consistent with the predicted size. Successful removal of the GST-tag was achieved by thrombin cleavage and confirmed with SDS-PAGE analyses and western blot analysis (Figure 8A). Recombinant peptide was successfully separated from the cleaved tag, using ion exchange chromatography, and desalted on a C8 column. Mass spectrometry revealed that the recombinant peptide had a size of 5.495 kDa, which matched the predicted mass (Figure 8B). Peptide mass fingerprinting confirmed that recombinant Vv-AMP1 resulted from the DNA sequence encoding for the mature Vv-AMP1 peptide.

### Antimicrobial activity of Vv-AMP1

**Figure 9:**
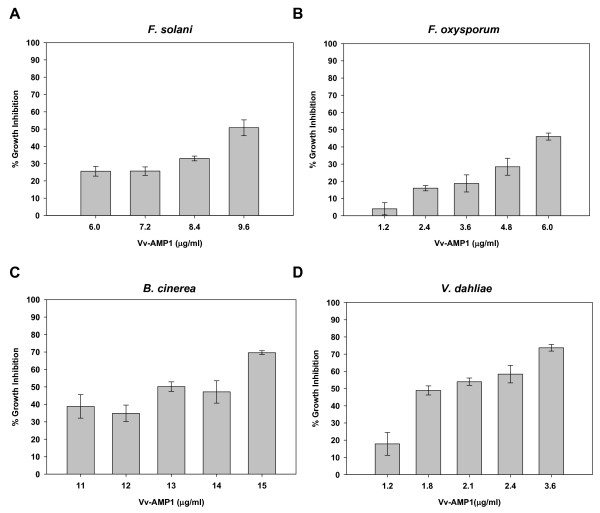
**Antifungal activity of Vv-AMP1 on a panel of plant pathogenic fungi**. Microspectrophotometric readings were recorded every 24 hours and compared to the untreated fungal controls. The data is represented as a percentage of fungal growth as compared to the untreated control reactions with no peptide. The experiment was repeated three times and the standard deviation for each reaction was less than 5%. Growth inhibition was determined after 48 hours of growth for the *Fusarium *and *B. cinerea *strains and after 72 hours for *V. dahliae*. The effect of Vv-AMP1 on the germination and growth of *F. solani *(**A**), *F. oxysporum *(**B**), *B. cinerea *(**C**) and *V. dahliae *(**D**) is shown.

Recombinant Vv-AMP1 was tested against several plant pathogenic fungi using a dose-response growth inhibition assay. The activity of Vv-AMP1 on fungal hyphae was assessed by incubating fungal spores in the presence of various concentration of Vv-AMP1 over a 72 hour period, with the IC_50 _value being determined after 48 hours of incubation (Figure 9A–D). Vv-AMP1 had a severe effect on the accumulation of fungal biomass over time in all of the fungal isolates tested and was most active against *F. oxysporum *(Figure 9B) and *V. dahliae *(Figure 9D), the two causal agents of wilting disease, with IC_50 _values of 6 μg/ml and 1.8 μg/ml, respectively. Vv-AMP1 was however less effective against *F. solani *with an IC_50 _value of 9.6 μg/ml (Figure 9A). The necrotrophic fungi *B. cinerea *(Figure 9C) was inhibited with an IC_50 _value of 13 μg/ml. Treatment of *B. cinerea *spores with peptide concentrations above 15 μg/ml resulted in > 95% growth inhibition, while a concentration of 30 μg/ml completely arrested spore germination (data not shown). The peptide showed no inhibition of *A. longipes*, even at peptide concentrations above 20 μg/ml (results not shown).

Microscopical analyses of fungal hyphae treated with Vv-AMP1 showed no signs of the characteristic hyperbranching effect associated with some plant defensins. Vv-AMP1 did, however, severely alter the ability of fungal hyphae to elongate and most hyphal tips had a swollen appearance. Granulation of the hyphal cytoplasm was also observed in most fungi treated with Vv-AMP1 (data not shown).

**Figure 10:**
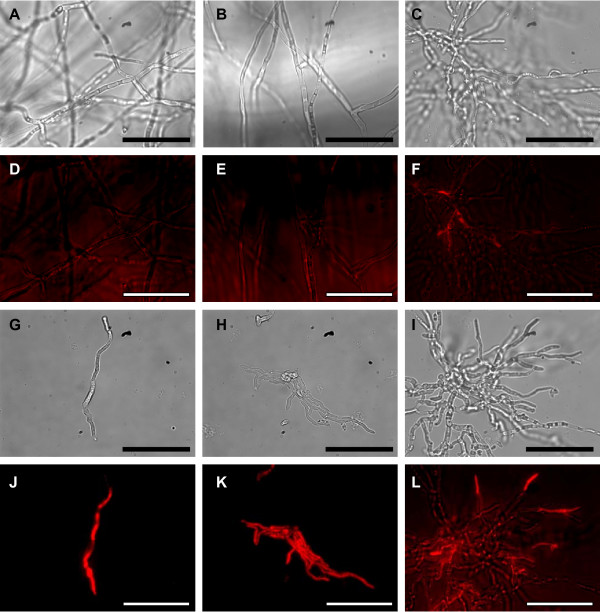
**Fluorescent microscope analysis of Propidium Iodide uptake during the membrane permeabilization assay**. (**A**-**C**), light microscope images and (**D**-**F**) Fluorescent images of untreated *F. oxysporum, F. solani and V. dahliae *hyphae respectively. (**G**-**I**), Light microscope images and (**J**-**L**) fluorescent images of Vv-AMP1 treated *F. oxysporum, F. solani and V. dahliae *hyphae respectively. Fungi were grown for 40 hours in the presence of Vv-AMP1 at peptide concentrations of 6 μg/ml for *F. solani*, 9.6 μg/ml for *F. oxysporum *and 1.8 μg/ml for *V. dahliae*. Afterwards fungal hyphae were stained with Propidium iodide for 10 min, washed with 1XPBS and subjected to fluorescent microscopic analysis. Bar = 50 μm

A propidium iodine treatment combined with fluorescent microscopy showed high levels of fluorescence present in the Vv-AMP1 treated samples when compared to the untreated fungi that showed no fluorescence (Figure 10). This is an indication that the fungal membranes were compromised by the presence of the Vv-AMP1 peptide. Fluorescence was observed throughout the affected hyphae in both *Fusarium *strains (Figure 10), whereas fluorescence was only present at the tips of Vv-AMP1 treated hyphae from *V. dahliae *(Figure 10L).

### Recombinant Vv-AMP1 is heat-stable and sensitive to proteinase activity in preliminary stability assessments

**Figure 11:**
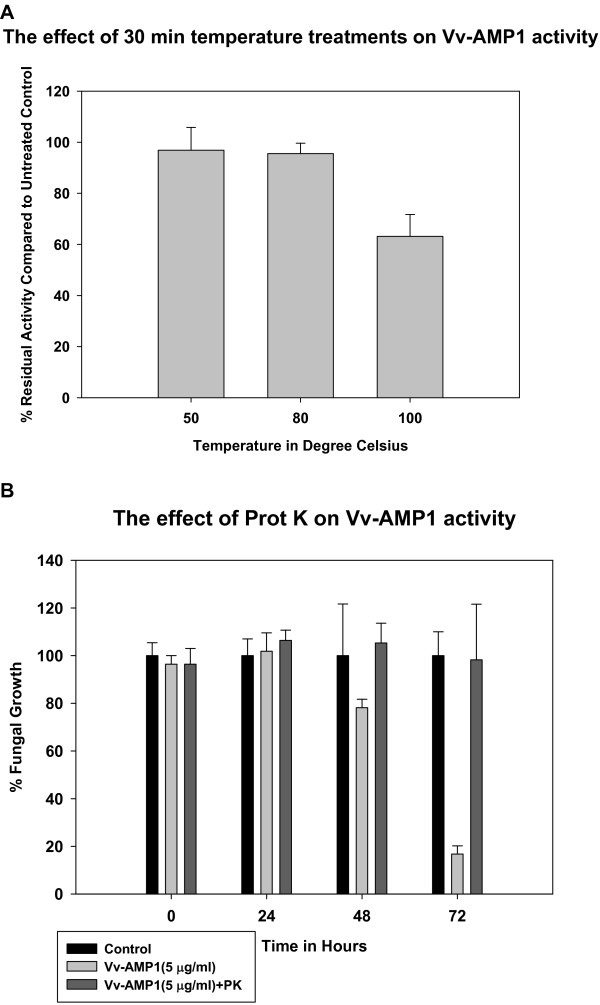
**Stability assessment of Vv-AMP1**. (**A**) Temperature stability of Vv-AMP1. After heat treatment the remaining antifungal activity of Vv-AMP1 was scored against the control with no heat treatment (25°C). (**B**) Stability of Vv-AMP1 against proteinase K (PK). The antifungal activity of Vv-AMP1 (at a dose of 5 μg/ml) was scored against *V. dahliae *after treatment with 100 μg/ml proteinase K at 37°C for 16 h.

Vv-AMP1 was tested for its stability at different temperatures using an antifungal growth assay against *Botrytis cinerea *(Figure 11A). Vv-AMP1 showed remarkable stability at temperatures up to 100°C. Ninety five percent of its antifungal activity was retained after 30 min of treatment at 80°C and 62% at 100°C for 30 min.

Vv-AMP1 was very sensitive to proteinase K treatment, confirming its proteinaceous nature. Enzyme treatment at 100 μg/ml totally abolished the activity of Vv-AMP1, as determined by an antifungal assay against *V. dahliae *(Figure 11B).

## Discussion

### The isolation and characterisation of a plant defensin encoding gene from grapevine

Plant defensins are small, cysteine-rich peptides with a basic nature that exhibit a broad spectrum of antimicrobial activity and have been implicated in the innate defense system of plants. Here we report the isolation and characterization of the first defensin peptide and its encoding gene from *Vitis vinifera*, the world's most important fruit crop. The 234 bp open reading frame isolated from *V. vinifera *berry cDNA (Figure 1) encoded Vv-AMP1, a peptide with significant consensus and structural homology to the peptide family of plant defensins. Analysis of the grapevine genome revealed that only one copy of *Vv-AMP1 *is present in the *Vitis vinifera *genome and the two hybridization signals observed in the Southern blot analysis are due to heterogeneity in the *Vv-AMP1 *locus. Moreover, the isolated gene is highly conserved within the *Vitis *genus (Figure 3). Most of the non-vinifera *Vitis *spp. that yielded homologous sequences are known for their high levels of natural resistance against fungal pathogens. Bioinformatical analyses on these sequences in comparison with the *V. vinifera *sequence showed high levels of homology and shared deduced structural features. The recently completed grapevine genome sequence revealed additional putative defensin sequences in *Vitis vinifera *(results not shown) that could be targeted for isolation and characterization as well.

Plant defensins exhibit an array of expression profiles and can be expressed constitutively, in a tissue specific pattern and also induced by various environmental stimuli [34,52,54-60]. The expression profiling of *Vv-AMP1 *showed that this gene is highly regulated. *Vv-AMP1 *showed a tissue-specific and developmentally regulated expression pattern, being only expressed in grape berry material (Figure 7A–B). Under the conditions tested, no additional inducers were observed and none overcame the berry-specific and developmentally regulated expression pattern (Figure 7C). The lack of response to wounding and infection by the known grapevine pathogen, *B. cinerea*, suggests that regulation of *Vv-AMP1 *expression is independent of the plant defense signalling pathways directed by these external stimuli. Hormone treatments in berries included SA, JA and ABA, but none of these showed any induction under the conditions tested. The expression pattern of *Vv-AMP1 *corresponds to berry ripening and it will be important to evaluate the effect of brassinosteroids further since it has recently been shown that these compounds act as inducers of berry ripening [61]. ABA is similarly linked to berry ripening, but did not have any effect on *Vv-AMP1 *expression levels. The specific expression pattern of this defensin gene is interesting and opposite to many genes linked to defence in grape berries. Transcriptome analysis and other studies have shown that many defence-related genes are down-regulated at the onset of berry ripening. It has been hypothesized that the defence mechanisms of the berry is not maintained during the post-véraison stages since the seed, containing the matured embryo, has obtained its hardened and protective seed coat when the berry starts to ripen. The plant has therefore completed its reproductive cycle and defence in berries are (from the perspective of the plant) less important. The signal peptide of Vv-AMP1 was shown to target GFP to the apoplastic regions when overexpressed in transgenic tobacco (Figure 6). Tissue-localization in the grape berries will provide further clues to the possible *in planta *role(s) of this peptide, specifically in relation to berry defence.

### Vv-AMP1: a highly stable and potent inhibitor of a range of fungal pathogens

#### Characteristic features of Vv-AMP1

Comparative analysis of the mature Vv-AMP1 with other members of the defensin family revealed that Vv-AMP1 shared the conserved amino acids present in the majority of plant defensins and was closest related to the defensin PPT from petunia (Figure 4). Classification studies, based on the precursor protein structure, grouped Vv-AMP1 with Class I of the defensin family [8]. Class I defensin peptides have a conserved precursor protein structure that consists of a N-terminal signal peptide followed by a mature defensin domain [8].

The Vv-AMP1 gene was successfully overexpressed in *E. coli *to yield purified peptide which could be subjected to accurate determination of size, as well as peptide fingerprinting. These results, as well as western blot analysis confirmed that the peptide was purified to homogeneity. The purified peptide was shown to be highly heat-stable, but readily deactivated by protease treatment. Preliminary results (not shown) indicates that Vv-AMP1 is quite sensitive to the divalent cation Ca^2+ ^when evaluated against *B. cinerea*. The sensitivity to cations is a characteristic well documented for many members of the plant defensin family [14,15], but is highly dependent on the fungal pathogen tested. Since the peptide is targeted to the apoplastic area, this indication of Ca^2+ ^sensitivity, as well as other cations, will have to be tested further against a broader range of fungi, specifically against the fungal pathogens of grapevine.

#### Inhibition profile and antifungal characteristics of Vv-AMP1

The panel of pathogens tested included various wilting disease pathogens as well as *Botrytis*, as a grapevine pathogen. Many of the grapevine fungal pathogens cannot be cultured *in vitro *(such as the powdery and downy mildew pathogens) and could not be included in this evaluation. Vv-AMP1 was especially effective against the causal agents of wilting disease, but also inhibited the necrotrophic *Botrytis*. Vv-AMP1 limited the fungal biomass of the test organisms, but did not induce typical morphological changes (under the conditions tested) in the treated cultures, classifying Vv-AMP1 as a non-morphogenic antifungal defensin peptide.

The inhibition profile of Vv-AMP1 is interesting and promising, both from the perspectives of general plant biotechnology, as well as understanding and manipulating grapevine defence specifically. The strong activity of Vv-AMP1 towards *V. dahliae *is of great interest in the engineering of disease resistant crops species. Vv-AMP1 is very active against *V. dahliae*, with particularly low IC_50 _values. A defensin peptide from *Medicago sativa*, alfAFP, has already showed great economical potential [62] by protecting potato against *V. dahlia *under field trial conditions. The activity of the peptide against grapevine pathogens still needs further evaluation, but preliminary inhibition analysis with a few pathogenic fungi that can be cultured *in vitro *yielded very promising results. Several of the grapevine trunk disease pathogens show strong sensitivity to the peptide *in vitro *(M. Tredoux, Institute for Wine Biotechnology, personal communication). Most of the economically important grapevine fungal pathogens require the plant host for growth and transgenic overexpression studies and whole plant infection assays are needed to determine the effect of the peptide on the these pathogens.

The observed activity of Vv-AMP1 was associated with alteration of fungal membrane permeability as indicated by the propidium iodide uptake assay and fluorescent microscopy (Figure 10). Previous studies have shown that plant defensins alter fungal membrane permeability, which is associated with a rapid uptake and efflux of Ca^2+ ^and K^+ ^[63,64]. It remains to be elucidated whether Vv-AMP1 used the same mechanism for the observed induction of membrane permeabilization.

Other questions that remain to be answered are the *in planta *stability of the peptide when overexpressed and the nature and degree of disease resistance that might be afforded by the peptide. The putative promoter of the *Vv-AMP1 *gene has also been isolated from grapevine (results not shown) and might provide valuable insights into the regulation of this defensin.

## Conclusion

*Vv-AMP1 *encodes for a berry specific, developmentally regulated peptide with homology to the superfamily of plant defensins. Mature Vv-AMP1 is directed to the apoplastic regions of the plant cell by a 30 amino acid signal peptide. Recombinant Vv-AMP1 showed a broad spectrum of antifungal activity at low concentrations against both necrotrophic and wilting disease causing fungi. Vv-AMP1 activity was classified as non-morphogenic and possibly associated with membrane permeabilization. The antifungal activities of the peptide are promising enough to merit further investigation of its potential in biotechnology approaches to increase fungal resistance in important crop species.

## Methods

### Plant materials and microbial strains

*Escherichia coli *strain DH5α were used for all cloning experiments, while *E. coli *strain BL21 (Rosetta-gami pLys S) DE3 (Novagen (Madison, WI, USA) was used for recombinant protein production. *Botrytis cinerea*, *Fusarium oxysporum *and *Fusarium solani *were obtained from the Department of Plant Pathology (DPP), Stellenbosch University. *Alternaria longipes *(ATCC 26293), *Fusarium oxysporum *(ATCC 10913) and *Verticillium dahliae *(ATCC 96522) were obtained from the American Type Culture Collection. All of the above fungal strains were maintained on potato dextrose agar at 25°C until sporulation. Tobacco seeds were obtained from Lehle Seeds, Round Rock TX 78681, USA and tobacco plants were maintained on Murashige Skoog (MS) medium [65] in a growth room with a temperature of 25°C and a 16 h photoperiod.

### Primer design and defensin gene isolation

The EST database of *V. vinifera *at TIGR [66] was screened using the BLAST algorithm. The database was screened with the γ-thionin sequence (PPT, gb|L27173.1) from *Petunia inflata*. Primers were designed from the EST clone TC69032 to recognize the complete coding sequence encoded within the EST.

Total RNA was isolated from 1 g *V. vinifera *cv. Pinotage root, leaf and berry tissue using a sodium perchlorate method [67]. cDNA was synthesized from total RNA using the SuperscriptIII cDNA synthesis kit (Invitrogen, Carlsbad, USA).

Genomic DNA was isolated from Pinotage leaves. Leaf tissue was collected, flash frozen in liquid nitrogen and ground to a fine powder. One gram of tissue was extracted with 10 ml of extraction buffer according to an established method [68].

The genomic and cDNA isolated from *V. vinifera *cv. Pinotage were used as templates in PCR strategies to isolate possible *Vitis *defensin sequences. The primer set used was, forward primer Vitisdef-5' (5'-GGCTCGAGATGGAAGGGCTCTCAACGTT-3') together with the reverse primer Vitisdef-3' (5'-CCGGATCCTTAACAATGCTTAGTGC-3'). PCR products obtained were cloned into the pGEM-T easy vector (Promega Corporation, Madison, USA) and sent for sequencing. Sequences obtained were analyzed using the BLAST algorithm and clones showing homology to the defensin family were termed *Vv-AMPs*.

### Southern blot analyses of *Vv-AMP1*

Genomic DNA was isolated from *V. vinifera *cv. Pinotage as described above. Pinotage genomic DNA was digested with *Kpn*I, *Spe*I or *Xba*I and separated on a 0.8% [w/v] agarose TAE gel. After transfer to a nylon membrane [69], the membrane was probed with a DIG-labeled cDNA probe of *Vv-AMP1*. Chemiluminescent detection was performed according to the DIG application manual for filter hybridization (Roche Diagnostics GmbH, Mannheim, Germany). Each hybridization signal represents a single copy of Vv-AMP1.

### Sequence analysis of *Vv-AMP1 *within the *Vitis *genus

Germplasm of other *Vitis *species were obtained from the USDA-ARS National Clonal Germplasm Repository (Davis, CA 95616, USA). These included genomic DNA for *V. afganista, V. x andersonii, V. aestivallis, V. cinerea *var. floridana, *V. labrusca *and *Ampelopsis aconitifolia *var. galabra. Genomic copies of *Vv-AMP1 *were isolated from the various germplasms using the same PCR based strategy to isolate the genomic copy of *Vv-AMP1 *from Pinotage. Isolated genes were cloned into the pGEM-T easy vector and sequenced. Genomic sequences obtained for the different *Vitis *species were analyzed with the AlignX software from the VectroNTI suite (Invitrogen, Carlsbad, USA) and final alignments were created in ClustalX [70].

### Expression pattern of *Vv-AMP1*

Total RNA was isolated from *V. vinifera *cv. Pinotage leaves, flowers and the different developmental stages of berry ripening. Tissue was collected and ground in liquid nitrogen to a fine powder. One hundred mg tissue from leaf, flower and green berry tissue was extracted with 800 μl extraction buffer at 65°C (2% [w/v] CTAB, 2% [w/v] PVP-40, 100 mM Tris/HCl (pH 8.0), 25 mM EDTA, 2.0 M NaCl, 2% [v/v] β-mercaptoethanol and 0.5 mg/ml Spermidine) for 5 min [71]. Total RNA from véraison to ripe berries was isolated with the sodium perchlorate method [67].

Total RNA was separated on a 1.2% [w/v] agarose formaldehyde gel (QIAGEN RNA/DNA handbook) and transferred to positively charged nylon membranes (Roche Diagnostics GmbH, Mannheim, Germany) [69]. Membranes were probed with a DIG-labeled *Vv-AMP1 *cDNA probe and Elongation factor1 alpha (*EF-1α*) gene from *V. vinifera *(TC65250) as internal standard. Pre-hybridization and hybridization were performed at 50°C. Chemiluminescent detection was performed according to the DIG application manual for filter hybridization (Roche Diagnostics GmbH, Mannheim, Germany).

For analysis of the chemical induction of *Vv-AMP1*, *V. vinifera *leaves were floated on 300 mM NaCl or 5 mM abscisic acid. Material was collected after 1, 6, 16 and 24 hours after each induction experiment and frozen in liquid nitrogen. Induction of *Vv-AMP1 *by wounding and *B. cinerea *infection was also assessed on leaf material. To evaluate the effect of wounding, leaves were subjected to mechanical damage and floated on distilled water in Petri dishes. Leaf material was collected and frozen in liquid nitrogen 1, 6, 16 and 24 hours after the initiation of the wounding experiment. *B. cinerea *infection was achieved by submerging leaves in a spore suspension of 50% grape juice containing 2000 spores/ml. After inoculation leaves were placed in Petri dishes under conditions of 100% relative humidity and incubated at room temperature. Material was collected 6, 12, 24 and 48 hours after inoculation and frozen in liquid nitrogen. Detached pre-véraison stage berries were also subjected to the same inductions, as well as to 10 mM salicylic acid and 5 mM jasmonic acid. All induction experiments were repeated three times, the material pooled and subjected to two separate RNA extractions. RNA isolation and Northern blot analysis were performed as described above.

### Bioinformatical analysis of the deduced amino acids sequence of *Vv-AMP1*

The deduced amino acid sequence of Vv-AMP1 was produced in VectorNTi and analyzed using the BLASTP algorithm. Homologous sequences identified were further aligned using ClustalX [70]. The deduced Vv-AMP1 sequence was also subjected to disulfide bridge analyses using DIpro [72]; secondary structure analysis as well homology modeling were done using the software package LOOPP @ CBSU version 3.0 [73]. PDB files obtained were further analyzed using the software package VMD (Visual Molecular Dynamics) and final images were rendered using POV-Ray. Sub-cellular localization directed by the Vv-AMP1 signal peptide was predicted on the Proteome Analyst Specialized Sub-cellular Localization Server (PA-SUB) [74]. Peptide mass prediction was done with the Expasy tool, PEPTIDE-MASS [75].

### Confirming the subcellular localization directed by the Vv-AMP1 signal peptide

In an effort to confirm the sub-cellular targeting predicted by PA-SUB, the signal peptide from Vv-AMP1 was fused to the Green Fluorescent Protein (GFP) reporter protein. The signal peptide from Vv-AMP1 was isolated by PCR using the primer set Vitisdef-5' and Vitis SP-3' (5'-AAGCTTAGCCTCAGCCACCATCGG-3') and cloned into pGEM-T easy vector to yield pGEM-VvSP. The signal peptide was excised from pGEM-VvSP with *Xho*I and *Hin*dIII and cloned into the restriction enzyme prepared plant expression vector pART27cassette, a pART27 vector [76] containing the expression cassette from pART7 cloned into the *Not*I sites of pART27. The construct was termed pART27-VvSP. A modified mGFP5 gene was prepared by PCR from the ABRC clone pLMNC92. The forward primer PIV2GFP5-5' (5'-CCAAGCTTGTAAGTTTCTGCTTCTACCTTTGA-3') and reverse primer PIV2GFP5-3' (5'-GCCTCTAGATTATTTGTATAGTTCATCCATGC-3') were used to PCR the GFP sequence from the plasmid pLMNC92. This GFP sequence lacked the N-terminal endoplasmic reticulum signal peptide as well as a C-terminal HDEL ER retention signal. The PCR product was cloned into pGEM-T easy vector to yield pGEM-GFP5. The GFP fragment was excised from pGEM-GFP5 with *Hin*dIII and *Xba*I and cloned into the restriction prepared pART27-VvSP vector to yield the plant expression vector pARTVvSP-GFP, which would allow for the expression of the Vv-AMP signal peptide fused to GFP, under control of the constitutive 35S cauliflower mosaic virus promoter. The pARTVvSP-GFP construct was transformed into *Agrobacterium tumefaciens *strain EHA105 via electroporation [77] and tobacco was transformed using a standard leaf disc method [78]. Plantlets were regenerated under kanamycin selection on MS medium and plantlets showing GFP fluorescence in their root system were identified by fluorescent emissions after GFP excitation on a dark reader (Clare Chemical Research, CO, USA). Hand sections were prepared from leaf petioles (untransformed controls as well as transgenic lines overexpressing GFP) and mounted in MS salt solution containing 40% [v/v] glycerol. GFP localization was visualized under an Olympus IX 81 inverted microscope. GFP excitation was achieved at 470 nm and fluorescent images were captured using the GFP filter cube of the CelliR^® ^camera and software system (Olympus Soft Imaging Solutions GmbH). All images captured were subjected to the CelliR^® ^background noise reducing filters at an intensity setting of 7.

### Recombinant production of Vv-AMP1

The pGEX-2T system (Amersham Biosciences, NJ, USA) was used for the recombinant production of Vv-AMP1 in *E. coli*. This system would allow for the production of mature Vv-AMP1 peptide fused to a GST-tag. pGEM-Vv-AMP1 served as template to prepare the mature Vv-AMP1 sequence by PCR. This was achieved with the primer set Vv1-GST-5' (5'-GGCCGGATCCAGGACCTGTGAGAGTCAGAGCCACCG-3') and Vitdef-3'. The resulting product was cloned into the pGEM-T easy vector and termed pGEM-GSTVv1 mature. The mature fragment was digested from pGEM-GSTVv1 with *Bam*HI and *Eco*RI and cloned into the *Bam*HI and *Eco*RI prepared pGEX-2T vector. Positive clones were sequenced and termed pGEX-Vv1.

pGEX-Vv1 was transformed into the BL21 (Rosetta-gami pLysS) DE3 (Novagen Madison, WI, USA) and positive colonies were selected by plating onto LB agar containing 50 μg/ml ampicillin, 12.5 μg/ml tetracyclin, 15 μg/ml kanamycin sulphate and 34 μg/ml chloramphenicol. A single colony was inoculated into 5 ml LB medium with antibiotics and grown overnight at 37°C. One ml preculture was inoculated into four 1 l Erlenmeyer flasks containing 400 ml LB medium with antibiotics and grown at 37°C with continuous shaking until an OD_600 _of 0.7. Expression of the GST-Vv1 fusion was induced with 0.4 mM IPTG for 5 hours at 22°C.

Bacterial pellets were collected from each flask by centrifugation and resuspended in 5 ml GST column binding buffer (10 mM Na_2_HPO_4_, 1.8 mM KH_2_PO_4_, 2.7 mM KCl, 140 mM NaCl_2_, pH7.6 and 4 mM PefaBloc from Roche Diagnostics GmbH, Mannheim, Germany) and frozen at -80°C. Cells were disrupted by repetitive freeze thaw cycles from liquid nitrogen to a 37°C water bath. Cell lysate was collected through centrifugation at 10 000 rpm and adjusted to 5 mM MgCl_2_. Lysate was treated with 10 units of DNase I (Roche Diagnostics GmbH, Mannheim, Germany) for 15 min at room temperature to reduce viscosity. Triton × 100 was added to a final concentration of 1% (w/v) and the bacterial cell lysate was cleared through centrifugation at 10 000 rpm for 15 min and passed through a 0.22 μM filter.

Recombinant protein was batch purified with Glutathione agarose resin. A 2 ml bed volume of Glutathione agarose4B (Sigma, St Louis, USA) was added to the filtered lysate and the recombinant peptide allowed to bind overnight on a rotor mixer. Unbound proteins were removed by washing twice with 10 ml GST binding buffer, followed by two washes of 10 ml of GST binding buffer containing 1% Triton × 100 to remove unspecific bound proteins. Bound recombinant GST-Vv-AMP1 peptide was eluted with 10 ml elution buffer (50 mM Tris-Cl, pH 8.0, 10 mM reduced glutathione). The N-terminal GST-tag was removed by thrombin digestion overnight at room temperature with 20 units of enzyme (Amersham Biosciences, NJ, USA). SDS-PAGE analysis was used to confirm the purity of the recombinant fusion protein after affinity chromatography and the complete removal of the N-terminal GST-tag by thrombin digestion.

The GST-tag was separated from mature Vv-AMP1 peptide using cation exchange chromatography on a SP sepharose column (Amersham Pharmacia Biotech). Samples containing the mature Vv-AMP1 peptide were pooled and loaded onto a Strata C8 Solid Phase Extraction column (Phenomenex, Torrance, CA, USA). Bound peptide was desalted by washing with 5 column volumes of dH_2_O containing 0.1% [v/v] TFA and eluted with 5 ml of 60% [v/v] acetonitrile containing 0.1% [v/v] TFA. Eluted peptide was freeze-dried, dissolved in distilled water at a final concentration of 100 μg/ml and stored at -20°C.

### Size determination and identification of heterologous Vv-AMP1

To confirm the purification of the Vv-AMP1-GST fusion protein and evaluate the cleavage of the GST-tag from Vv-AMP1, 2 μg purified protein was separated on a 15% [w/v] Tris-Tricine gel [79]. After separation the gel was microwave stained in staining solution (Coomassie R250 in 50% [v/v] ethanol, 10% [v/v] acetic acid). The gel was destained with 12.5% [v/v] isopropanol and 12% [v/v] acetic acid. The exact size of mature Vv-AMP1 peptide samples was determined by LC/MS analysis on a Waters API Q-TOF Ultima instrument.

Identification of the peptide was achieved by peptide mass fingerprinting. Forty five μg recombinant Vv-AMP1 peptide was digested with the ProteoExtract^tm ^all-in-one trypsin digest kit (Calbiochem, La Jolla, CA, USA) and subjected to LC/MSMS analysis on a Waters API Q-TOF Ultima instrument. The resulting peaks were analyzed with the Expasy program FindPep [75].

### Preparation of antibody and immunoblotting

Polyclonal antibodies against Vv-AMP1 were produced in mice by immunizing three mice with 300 μg of the GST-Vv-AMP1 fusion protein in Freund's complete conjugate. Two booster injections consisting of 100 μg protein in Freund's complete conjugate were given at 2 week intervals and a final injection with 100 μg purified Vv-AMP1 was given 2 weeks before the terminal bleed.

Western blot analysis was conducted on 400 ng purified Vv-AMP1. The peptide was separated on a 15% (w/v) Tris-tricine gel [79] together with a low molecular weight marker (Sigma, St Louis, USA). One half of the gel was stained with Coomassie R250 and the other half electroblotted to a PVDF membrane (BioRad, Hercules, CA, USA). The membrane was blocked for 3 hours in blocking buffer (phosphate buffered saline, 0.1% [w/v] Tween 20 and 5% [w/v] skim milk) before incubating overnight in a 1:500 dilution of primary antibody prepared in blocking buffer. Detection of Vv-AMP1 was achieved with anti-mouse IgG secondary antibody and the ECL chemiluminecent system according to Amersham Biosciences, NJ, USA.

### Antimicrobial activity of recombinant Vv-AMP1

Quantitative antifungal activity of Vv-AMP1 was measured by microspectrophotometry [80]. The assay was preformed in a 96-well microtiter plate (Bibby Sterilin Ltd, Stone, Staffs, UK), where each well contained 100 μl half strength Potato Dextrose Broth (PDB) with 2000 fungal spores and purified Vv-AMP1 peptide of 1–20 μg/ml, respectively. Control reactions contained no peptide. Plates were incubated in the dark at 25°C for 3 days. Microspectrophotometric readings were taken every 24 hours at *A*_595_. All readings were corrected by subtracting the time zero readings from the time 24, 48 and 72 hour readings. Vv-AMP1 activity was scored after 48 hours and expressed in terms of % growth inhibition. Percentage growth inhibition is defined as 100× the ratio of the corrected *A*_595 _of the control minus the corrected *A*_595 _of the sample over the corrected *A*_595 _of the control. Each activity assay was independently repeated three times with three technical repeats per measurement. Microscope images were also collected directly from the antifungal assays with an Olympus IX70 inverted microscope. Images were captured with the Analysis^® ^software (Olympus Soft Imaging Solutions GmbH – results not shown).

The ability of Vv-AMP1 to cause fungal membrane permeabilization was assessed using a Propidium Iodide (PI) uptake assay [81], conducted on *F. oxysporum, F. solani *and *V. dahliae*. The permeabilization assay consisted of 200 μl half-strength PDB containing fungal spores (2 × 10^4 ^spores/ml) and Vv-AMP1 peptide at concentrations of 6 μg/ml for *F. solani*, 9.6 μg/ml for *F. oxysporum *and 1.8 μg/ml for the *V. dahliae *isolate. Fungal strains were incubated at 25°C in the presence of Vv-AMP1 for 40 hours. Control samples contained no Vv-AMP1. After incubation the samples were washed with 1xPBS and stained for 10 min in PI staining solution (25 μg/ml PI in PBS). Stained samples were washed twice with 1× PBS and viewed under an Olympus IX 81 inverted fluorescent microscope. Images were capture with the CelliR^® ^digital camera and software system (Olympus Soft Imaging Solutions GmbH). The presence of fluorescence is indicative of a compromised fungal membrane.

### Heat stability assessment and proteinaceous nature of recombinant Vv-AMP1

The stability of the heterologous Vv-AMP1 peptide was assessed by an antifungal assay as described above. The heat stability of the peptide was assessed at a final peptide concentration of 20 μg/ml against *B. cinerea *spores, with the peptide being pretreated at 25°C, 50°C, 80°C and 100°C for 30 min, before commencing with the antifungal assay. The activity of Vv-AMP1 was scored against the control reaction conducted at 25°C. Vv-AMP1 peptide was also subjected to a proteinase stability assay. Vv-AMP1 was treated with proteinase K (100 μg/ml) at 37°C for 16 hours. After digestion Vv-AMP1 was subjected to an antifungal assay against *V. dahliae *at a final concentration of 5 μg/ml. The activity of Vv-AMP1 was scored against a control reaction containing proteinase K (100 μg/ml), but without any Vv-AMP1 peptide added. All the above mentioned assays were done in triplicate.

## Authors' contributions

MAV supervised the work and helped with conceptual design and manuscript preparation as well as final data analysis. AdB performed conceptual and experimental design and was responsible for all the research procedures.
